# Guidance for creating individual and batch latinized binomial virus species names

**DOI:** 10.1099/jgv.0.001800

**Published:** 2022-12-02

**Authors:** Thomas S. Postler, Luisa Rubino, Evelien M. Adriaenssens, Bas E. Dutilh, Balázs Harrach, Sandra Junglen, Andrew M. Kropinski, Mart Krupovic, Jiro Wada, Anya Crane, Jens H. Kuhn, Arcady Mushegian, Jānis Rūmnieks, Sead Sabanadzovic, Peter Simmonds, Arvind Varsani, F. Murilo Zerbini, Julie Callanan, Lorraine A. Draper, Colin Hill, Stephen R. Stockdale

**Affiliations:** ^1^​ Department of Microbiology & Immunology, Vagelos College of Physicians & Surgeons, Columbia University Irving Medical Center, New York, NY 10032, USA; ^2^​ Consiglio Nazionale delle Ricerche, Istituto per la Protezione Sostenibile delle Piante, Sede Secondaria di Bari, 70126 Bari, Italy; ^3^​ Quadram Institute Bioscience, Norwich Research Park, Norwich, NR4 7UQ, UK; ^4^​ Institute of Biodiversity, Faculty of Biological Sciences, Cluster of Excellence Balance of the Microverse, Friedrich Schiller University Jena, 07743 Jena, Germany; ^5^​ Theoretical Biology and Bioinformatics, Department of Biology, Utrecht University, 3584 CH Utrecht, Netherlands; ^6^​ Veterinary Medical Research Institute, H-1143 Budapest, Hungary; ^7^​ Institute of Virology, Charité – Universitätsmedizin, corporate member of Free University Berlin, Humboldt-University Berlin, and Berlin Institute of Health, 10117 Berlin, Germany; ^8^​ Departments of Pathobiology, University of Guelph, Guelph, Ontario, N1G 2W1, Canada; ^9^​ Institut Pasteur, Université Paris Cité, CNRS UMR6047, Archaeal Virology Unit, 75015 Paris, France; ^10^​ Integrated Research Facility at Fort Detrick, National Institute of Allergy and Infectious Diseases, National Institutes of Health, Frederick, MD 21702, USA; ^11^​ Division of Molecular and Cellular Biosciences, National Science Foundation, Alexandria, VA 22314, USA; ^12^​ Latvian Biomedical Research and Study Centre, Riga, LV-1067, Latvia; ^13^​ Department of Biochemistry, Molecular Biology, Entomology and Plant Pathology, Mississippi State University, Mississippi State, MS 39762, USA; ^14^​ Nuffield Department of Experimental Medicine, University of Oxford, Peter Medawar Building, Oxford, OX1 3SY, UK; ^15^​ The Biodesign Center for Fundamental and Applied Microbiomics, School of Life Sciences, Center for Evolution and Medicine, Arizona State University, Tempe, AZ 85287-4701, USA; ^16^​ Structural Biology Research Unit, Department of Integrative Biomedical Sciences, University of Cape Town, Cape Town, South Africa; ^17^​ Departamento de Fitopatologia/BIOAGRO, Universidade Federal de Viçosa, Viçosa, MG 36570-900, Brazil; ^18^​ APC Microbiome Ireland, University College Cork, Cork, T12 YT20, Ireland

**Keywords:** binomial, ICTV, International Committee on Taxonomy of Viruses, Latinization, species name, virus nomenclature, virus taxonomy

## Abstract

The International Committee on Taxonomy of Viruses recently adopted, and is gradually implementing, a binomial naming format for virus species. Although full Latinization of these names remains optional, a standardized nomenclature based on Latinized binomials has the advantage of comparability with all other biological taxonomies. As a language without living native speakers, Latin is more culturally neutral than many contemporary languages, and words built from Latin roots are already widely used in the language of science across the world. Conversion of established species names to Latinized binomials or creation of Latinized binomials *de novo* may seem daunting, but the rules for name creation are straightforward and can be implemented in a formulaic manner. Here, we describe approaches, strategies and steps for creating Latinized binomials for virus species without prior knowledge of Latin. We also discuss a novel approach to the automated generation of large batches of novel genus and species names. Importantly, conversion to a binomial format does not affect virus names, many of which are created from local languages.

## Introduction

Since their introduction by Carl Linnaeus in 1753, binomials in the general format *Genus_name species_epithet* (e.g. *Escherichia coli *) have become the officially preferred, universally recognized and adopted format for naming species in cellular biology (i.e. bacteriology, botany, mycology and zoology) [1–4]. These Latinized binomials remain unchanged across different languages, making them recognizable to an international readership; for example, the name for the species established for humans is ‘*Homo sapiens*’ in Arabic, Chinese, English and all other languages and thus can be easily recognized by the global community, whereas the noun ‘human’ occurs only in English. Also, as in the example above, Latinized binomials are often distinct from organism names. This difference is helpful in maintaining the logical distinction between a taxon (e.g. the species *Canis lupus*, which includes all wolves and describes the concept of a wolf) and the physical members of the taxon (e.g. individual wolves). Many aspects of Linnaean nomenclature have stood the test of time: for example, taxonomic names are typically expressed in Latin and typically built from Latin and Ancient Greek roots. Aside from providing stability in practice, there are additional advantages to the use of Latin. As a language without living native speakers, Latin may be less likely to incur the charge of cultural favouritism than many contemporary languages. Furthermore, stems derived from Latin and Ancient Greek are already widely used in the language of science across the world.

In virus taxonomy, names of taxa above the rank of species are already Latinized, following the biological format [5]. However, virus species names have not, until recently, followed a consistent format, setting virology apart from other systems of biological nomenclature. Frequent homonymy of virus species and virus names, currently still abundantly present in virus taxonomy, is another issue that may generate confusion [6–8].

In 2021, the International Committee on Taxonomy of Viruses (ICTV) changed the International Code of Virus Classification and Nomenclature (ICVCN) to mandate a binomial format for naming viral species similar to that used for cellular organisms [9, 10]. This new binomial format consists of a Latinized genus name and a ‘freeform’ species epithet [9, 10] but may include any text or numbers. During discussions preceding this change, reservations were voiced over mandatory Latinization of species epithets because converting established virus species names to a Latinized binomial format and even more so the creation of species names *de novo* might prove complex and time-consuming [11, 12]. To evaluate this concern, one of us (T.S.P.) converted 175 official species names in the order *Mononegavirales* and the family *Arenaviridae* to Latinized binomials [13]. This exercise revealed a practical ease of conversion and was subsequently expanded to include a larger set of species for negative-sense RNA viruses (≈650), which took approximately 2 weeks to complete.

Three conclusions could be drawn from these exercises: (i) a single individual could convert a considerable number of virus species names with limited time and effort; (ii) when evaluating these hypothetical new species names, ICTV Study Groups made some suggestions for alternatives but did not object to the majority of names, indicating that a large-scale conversion to Latinized binomials is unlikely to prove objectionable to the virology community; and (iii) conversion of current virus species names to Latinized binomials can be achieved in a formulaic fashion that does not require knowledge of Latin.

The recent changes to the ICVCN (taxonomic proposal [TaxoProp] 2018.001 G.R.binomial_species [14]) require submission of TaxoProps to change all established non-binomial species names to binomials by 2023 and all novel species names to be created in the binomial format starting in 2021 [9, 10]. Remaining optional is Latinization of species epithets. (An epithet is the second component of a binomial species name.) Indeed, an increasing number of species names has already been converted, and many Study Groups adopted Latinization efficiently. (For the current ICTV taxonomy, see https://ictv.global/taxonomy and the various ICTV Virus Taxonomy Profiles and linked ICTV Report chapters published by *The Journal of General Virology* at https://www.microbiologyresearch.org/content/ictv-virus-taxonomy-profiles, as well as taxonomy updates published by *Archives of Virology's* Virology Division News at https://link.springer.com/journal/705/topicalCollection/AC_d2e3a8e46d7ae45cb31eec24c4b7d987). Here, we provide guidelines for generating Latinized binomial virus species names to assist virologists and other scientists interested in adopting this particular format. As high-throughput sequencing is revealing many thousands of new viruses that will eventually be classified [15–27], species names will need to be created on a massive scale. Thus, we also discuss an algorithmic approach to the generation of linguistically correct Latinized binomials. Similar approaches have already led to the creation of more than a million hypothetical names for species of *Archaea* and *Bacteria* [28, 29], and have been recently implemented for close to 1000 novel prokaryotic virus species names [9, 30]. Therefore, Latinized binomials offer a viable alternative to other free-form names for individuals and ICTV Study Groups proposing new species through TaxoProps.

## A step-by-step guide to the creation of individual latinized binomial virus species names

### Grammatical background

Linnaean binomials consist of two italicized words, a capitalized genus name (e.g. *Homo:* a human), followed by a species-specific descriptor in the lower case, known as a species epithet (e.g. *sapiens*: wise, judicial or rational). Together, these two words constitute the species name. Names are built using letters from the Medieval (aka ISO basic) Latin alphabet, while avoiding use of diacritical marks and other characters, aside from hyphens (ICVCN Rule 3.13). The genus name is a noun or an adjective used as a noun. The species epithet is (i) an adjective that describes the genus (e.g. *Homo sapiens*: the wise human); (ii) a noun in the genitive case (i.e. indicating a ‘pertaining to’ relationship of the species epithet to the genus name) (e.g. *Borrelia burgdorferi *, a bacterial species in the genus *Borrelia* named after its discoverer Wilhelm Burgdorfer); or (iii) a noun in the nominative case used in apposition (i.e. as an additional description of the taxon) (e.g. *Tyrannosaurus rex*, which means ‘tyrant lizard, the king’).

When an adjective is used as the species epithet, it assumes the same grammatical gender as the associated genus name. This has sometimes created problems when those not expert in Latin try to create names in other systems of nomenclature. However, as all virus genus names (e.g. *Betacoronavirus*) uniformly end in the suffix *-virus* (derived from a Latin word, neuter in grammatical gender, meaning ‘poison’), all virus genus names and adjectival species epithets are treated as neuter. Similarly, the ICTV also considers the genus suffixes -*satellite*, -*viriform*, and -*viroid* neuter. Hence, all recommendations listed here may be applied to satellite, viriform, viroid and virus species naming. This uniformity simplifies the use of Latinized virus species epithets when compared to other biological taxonomies, in which all three grammatical genders are amply represented among genus names. However, Latin adjectives come in a variety of different classes with different endings, known as declensions, and it is not always immediately obvious which one is the correct one to use. Therefore, we recommend that virologists without Latin knowledge only use adjectives for species epithets that refer to the geographical location, which can be constructed in a formulaic manner as described below.

Forming the genitive case of a Latin noun is typically less complex by comparison, as each Latin noun has exactly one singular genitive form, which is listed in standard Latin dictionaries. Those available for free use online include the aptly named ‘Latin Dictionary’ (https://www.online-latin-dictionary.com) and ‘William Whitaker’s Words’ (https://latin-words.com ).

### Names based on geographical location

**Fig. 1.**
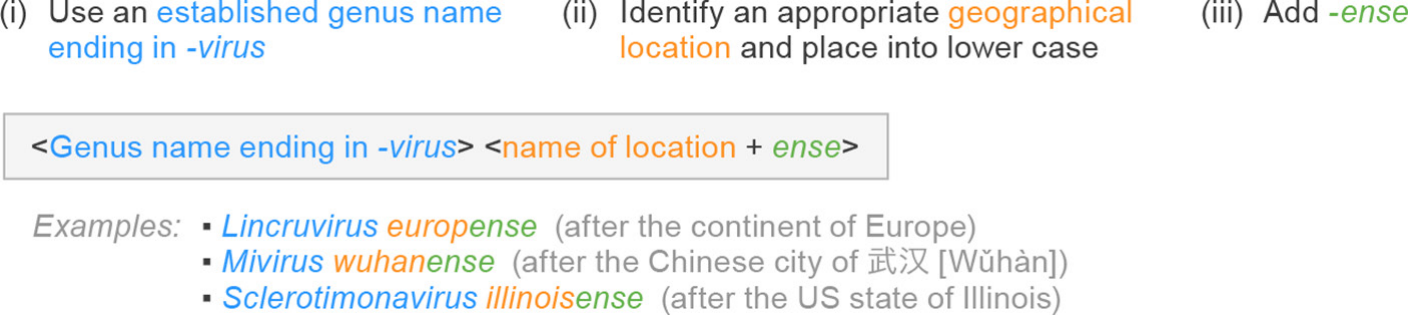
Latinized binomial virus species name formation based on geographical locations. Examples are ICTV-ratified, official species names.

The Latin suffix *-ensis* denotes an adjective that describes a location. This suffix is commonly used in other biological taxonomies; e.g. *Homo neanderthalensis* is the name of the species for hominins originally found in the Neandert(h)al (Neander Valley), Germany. Because viruses are often named after the geographical locations at which they were first discovered or sampled, this suffix offers a convenient method of creating grammatically simple, neuter adjectives to serve as species epithets, without the need to identify the declension. The neuter form of -*ensis* is *-ense*, which can be directly attached to the name of a location. For instance, *Examplovirus neanderthalense* could be a species in the fictitious genus *Examplovirus* for a virus in some way associated with the Neandert(h)al region. Analogously, the related species *Examplovirus bostonense* would be associated with the city of Boston. Of note, the nature of species epithets does not require them to be unambiguous descriptors of a unique location (or anything else), as long as they are unambiguous descriptors of the virus species. Indeed, there are up to 34 municipalities named Boston around the world; yet it is immaterial to which one of these *bostonense* is referring, as long as there is no other *Examplovirus* species bearing the same epithet. When the suffix *-ense* is added to a word ending with a vowel, that vowel may be omitted for aesthetic reasons, but, for consistency and simplicity, we recommend retaining these vowels (e.g. *Examplovirus atlantaense*, not *Examplovirus atlantense*) except if a vowel is repeated (e.g. *Examplovirus zairense*, not *Examplovirus zaireense*) (Fig. 1).

### Names based on persons, objects, organizations or institutions

**Fig. 2.**
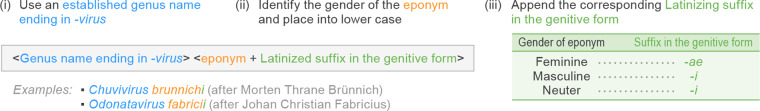
Latinized binomial virus species name formation based on persons, objects, organizations or institutions. Examples are ICTV-ratified, official species names.

To create a Latinized species epithet based on the name of a person, object, organization or institution, the name may be Latinized and converted to the genitive form. Latinization of personal names is typically achieved by appending the appropriate Latin suffix. The ending differs for the three grammatical genders, with the base form being *-a* for feminine (e.g. a female person; woman), *-us* for masculine (e.g. a male person; man) and *-um* for neuter (e.g. an object, organization or institution). For each of these, the cognate genitive form can be created using the endings provided in Fig. 2. For example, a virus species in the fictitious genus *Examplovirus* named after Jane Goodall, a woman (Goodalla), might be called *Examplovirus goodallae*, whereas a related species named after Max Delbrück, a man (Delbruckus), could be designated *Examplovirus delbrucki* (Fig. 2). Further, a species to be named after UNICEF, an organization (unicefum), could be designated *Examplovirus unicefi*. These guidelines describe the same simple approach also used in zoology, but it should be noted that other taxonomical frameworks in biology access a greater diversity of options in the formation of eponyms. For example, the interested reader is referred to the International Code of Nomenclature of Prokaryotes [3]. We do not discuss these broader possibilities further for the sake of simplicity.

### Names based on a disease

**Fig. 3.**
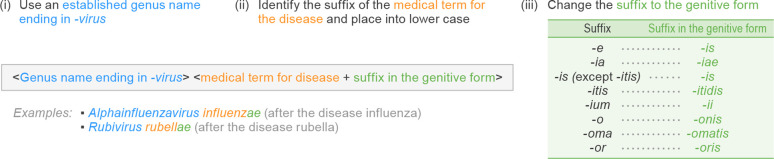
Latinized binomial virus species name formation based on diseases. Examples are ICTV-ratified, official species names.

Generating a Latinized species epithet based on a disease caused by a member virus is often convenient, as medical nomenclature typically uses Latin or Latinized suffixes in disease nomenclature as well. To form the genitive of such a disease name, only the suffix has to be altered. Fig. 3 provides a list of suffixes commonly used in medical terminology and the corresponding genitive forms. For instance, if a member of a species in the fictitious genus *Examplovirus* causes an inflammation of the tonsils (i.e. tonsillitis), the species name could be *Examplovirus tonsillitidis*. Of note, medical terminology is frequently an amalgam of Latin and Greek etymologies, which occasionally affects suffixes. Nonetheless, Latin-based declension (even of Greek suffixes) is recommended wherever possible (Fig. 3).

### Names based on the host

All established organismal species, and therefore almost all virus host species, are already named using Latinized binomials. Consequently, creating a Latinized virus species epithet based on a host taxon only requires finding the genitive form of that taxon. However, this approach should only be taken if the host taxon is well established and considered to be stable, as confusion will inevitably arise if a host taxon name is updated but the virus species name is not. In general, we recommend choosing the genus name of the host, rather than its species epithet, because the epithet by itself is typically less informative and many viruses infect hosts of several species within the same genus. As with geographical names, however, complete accuracy in the choice of host-based species epithets is not required (or even realistic), as long as the resulting binomial is an unambiguous descriptor of the virus species.

**Fig. 4.**
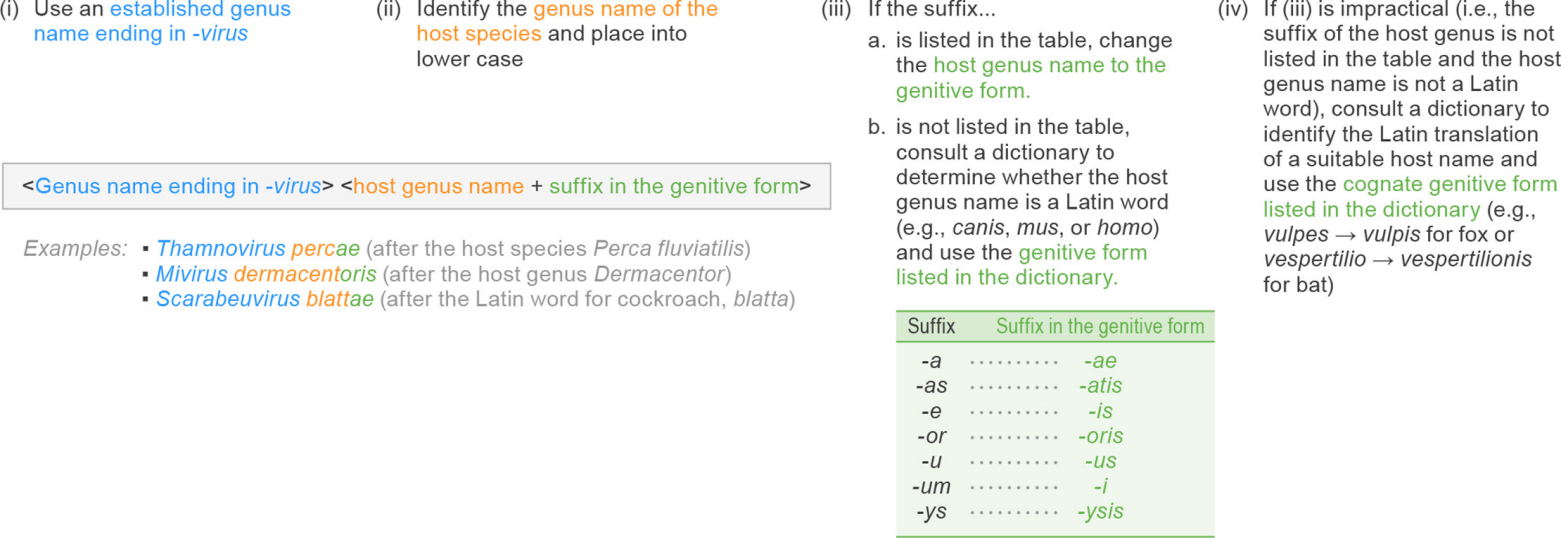
Latinized binomial virus species name formation based on virus hosts. Examples are ICTV-ratified, official species names.

Fig. 4 lists Latin suffixes commonly used in the naming of non-viral genera. This list is by necessity incomplete, as some suffixes are used by multiple different declensions. In those cases, different words with the same ending in the base form (the nominative case) may have different endings in the genitive form. Therefore, for such a situation, we recommend that investigators use a Latin dictionary or other credible resource to determine if the host genus name is a Latin word (e.g. *Homo*) and, if so, to identify the corresponding genitive form. If the genus name is not a Latin word, we suggest that investigators use the same dictionary or resource to ascertain the Latin translation, along with the cognate genitive form, of the common name of the host instead. For instance, for a species included in the fictitious genus *Examplovirus* whose members infect Darwin’s foxes (*Lycalopex fulvipes*), a quick search would reveal that the Latin word for fox is *vulpes*, with the genitive *vulpis*. Thus, this virus species might be named *Examplovirus vulpis*.

Other rules apply if a virus species is to be named after a host taxon ranked above genus, as such taxa are by convention always denoted in the plural form. This situation is grammatically more complex, as the only unambiguous suffix is *-ae* (e.g. Hominidae, the family that includes humans). In such cases, the singular genitive form is also *-ae*. An examplovirus species whose members infect hominids might thus be named *Examplovirus hominidae*. An examplovirus species for a virus isolated from bats, which comprise the entire taxonomic order Chiroptera, might be named *Examplovirus vespertilionis*, as the Latin word for bat is *vespertilio* (Fig. 4).

### Contractions and omissions

Established non-Latinized virus species names and virus names often consist of more than two words, precluding a direct conversion to Latinized binomials. Virological taxonomy has a long history of elegantly circumventing this problem by use of contractions, as exemplified by the family names *Tombusviridae* (after tomato bushy stunt virus) or *Hepeviridae* (after hepatitis E virus). We recommend continuing this tradition when creating Latinized binomial species names. For instance, the virus species currently named *Britarnavirus 2* could be renamed *Britarnavirus britcolense*, as the original isolation of the member virus, marine RNA virus BC-2, occurred in British Columbia. Similarly, *Mamastrovirus 8* could be converted to the Latinized binomial *Mamastrovirus homustovis*; the original isolates of this species were named HMO for their similarity to human (genus *Homo*), mink (genus *Mustela*) and sheep (genus *Ovis*) astrovirids. If contractions are not practical but an investigator wants to preserve a relationship to an established, non-Latinized species name or virus name, parts of that name could be Latinized and others omitted. For example, the species formerly designated as *Wheat American striate mosaic cytorhabdovirus* was recently renamed *Cytorhabdovirus tritici*, as wheat belongs to the genus *Triticum*.

### Creative approaches

The information provided here is intended to offer practical guidelines for the creation of Latinized virus species epithets that are applicable to a majority of use cases. However, there will be instances in which these guidelines are insufficient. In such cases, we encourage investigators to use their imagination to create their own Latinized alternatives derived with the guidelines above. For instance, the species designated as *Severe acute respiratory syndrome-related coronavirus* [currently the taxonomic home of both severe acute respiratory syndrome coronavirus (SARS-CoV) and SARS-CoV-2] could be converted to the arguably much simpler *Betacoronavirus sarscovi*, using the acronym SARS-CoV and Latinizing it as a neuter eponym. Analogously, if SARS-CoV-2 were to be assigned to a separate species, consultation of a Latin dictionary would reveal that the Latin word for ‘second’ is *secundus*, which could be used in a contraction with *sarsi* to create the Latinized binomial *Betacoronavirus secusarsi*.

Other situations may require more creative approaches. For instance, there is no Latin word for echinoderm. However, echinoderms exhibit radial, often star-like morphology. The Latin word *radiale* approximately means ‘like the spokes of a wheel’. Following these considerations, as well as the guidelines provided above, the species *Echinoderm berhavirus* was recently renamed *Berhavirus radialis*.

Problems could arise when different species in the same genus have highly similar name-giving characteristics. For example, Antarctic penguin viruses A, B and C, which are members of the species currently named *Avian orthoavulavirus 17*, *18* and *19*, respectively, were all isolated at the same location, Kopaitic Island. This island was named after Boris Kopaitic O’Neill. Consequently, *Avian orthoavulavirus 17*, *18* and *19* could conceivably be renamed *Orthoavulavirus borisense*, *Orthoavulavirus kopaiticense* and *Orthoavulavirus oneillense*. Numbers and letters could also be incorporated into new species names. For instance, the former species *Trifolium pratense cytorhabdovirus A* and *Trifolium pratense cytorhabdovirus B* have been renamed *Cytorhabdovirus alphatrifolii* and *Cytorhabdovirus betatrifolii*, respectively.

### Case study for batch virus species name formation

Our recommendations focus primarily on the creation of Latinized virus species names on an individual basis. The current total number of established virus species (10 434; Master Species List #37, 2022 [31]) and the deadline to submit TaxoProps to accomplish the binomial conversion (2023) [9] is not prohibitive to a one-by-one approach [13]. The number is indeed a fraction of the more than a million species assigned in the rest of biology, each bearing a Latinized binomial name. However, the recent rapid increase in the discovery of novel viruses [15–27] presages an impending need to classify hundreds or thousands of viruses into novel species annually and to create corresponding binomial names on a similar scale.

We recently demonstrated that rapid batch formation of Latinized binomial virus species names is possible and acceptable to the ICTV. A thorough analysis of various metagenomic studies revealed a large number of relatives of the four classified positive-sense RNA viruses known to infect prokaryotes in 2020 [30]. This discovery required a fundamental taxonomic reorganization of the very small class *Allassoviricetes* (one order including one family with two genera and four species), leading to a now-ICTV-accepted, renamed class (*Leviviricetes*) that includes two orders, six families, 420 genera and a total of 883 species [9, 30].

Within this reorganized taxonomy, genus names for previously isolated and laboratory-studied bacterial RNA viruses were created manually, such as *Emesvirus* for the genus containing the well-known bacteriophage MS2, *Qubevirus* for bacteriophage Qβ or *Pepevirus* for bacteriophage PP7. Similarly, species epithets were also devised manually by utilizing different features of the isolated viruses. For instance, *Emesvirus zinderi* was named after Norton Zinder who discovered the first RNA phage; *Qubevirus durum* was named after the remarkable stability (durum means hardy) of the capsid of its member virus; and *Apeevirus quebecense* was named after the location of initial virus isolation. However, 871 metagenome-derived viruses, which lacked any known physical characteristics, required overcoming three hurdles: batch formation of genus names (which are necessary components of binomial species names), batch formation of species epithets and the unique combination of the two.

**Fig. 5.**
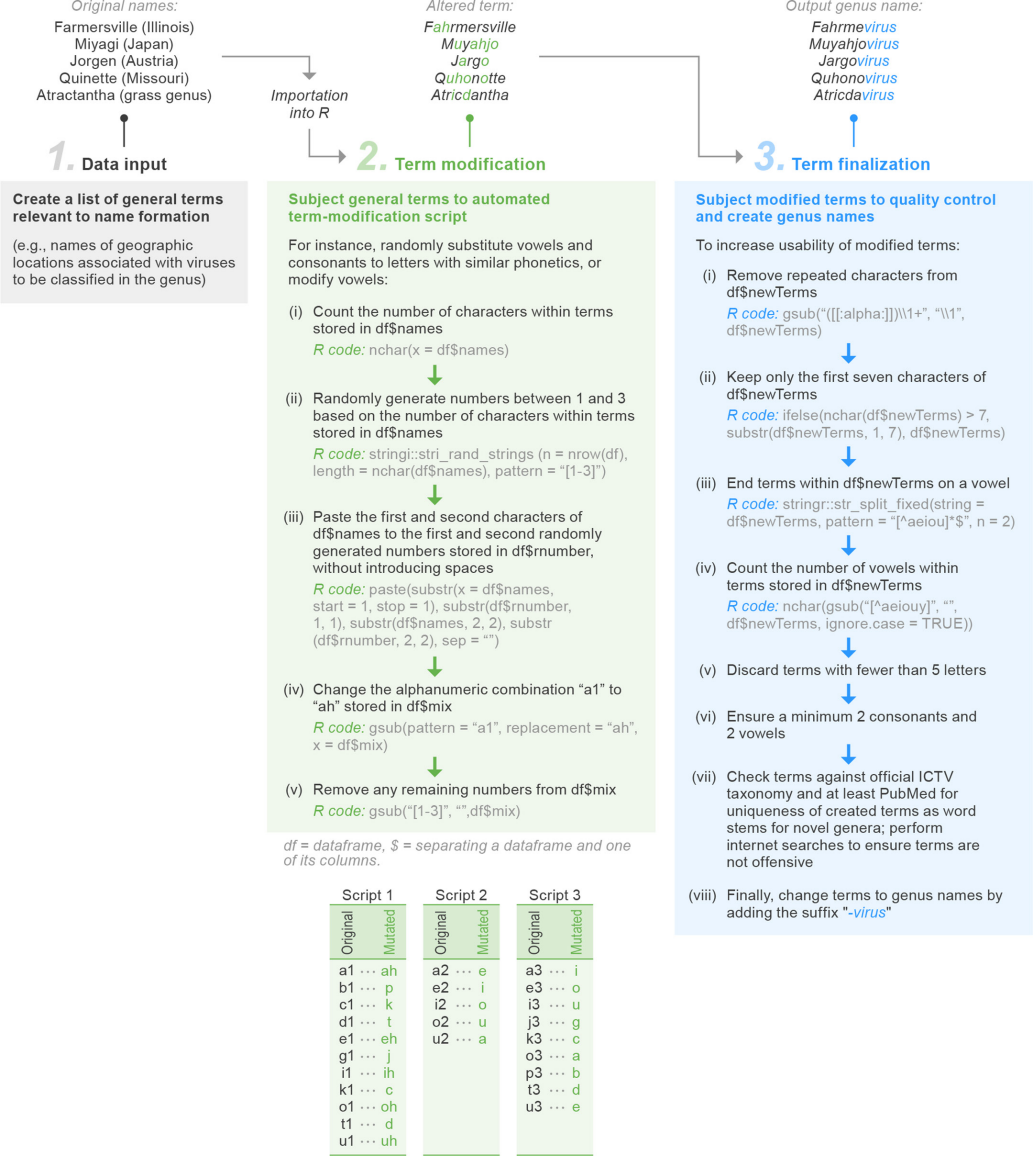
Example strategy for batch formation of virus genus names.

The formation of genus names followed a three-step process (Fig. 5). First, lists of terms were populated with basic data associated with the newly discovered viruses and pasted into comma-separated value fields for importation into R [32]. To mitigate any potential negative connotations that may arise when species were named after a person, place or object, a devised mutation of the assembled list of terms (also known as ‘strings’) was performed using R code; the terms were mutated using ‘gsub’ functions. A specific seed was set for reproducibility; before each letter of each term to be mutated the numbers 1, 2 or 3 were randomly assigned using the ‘stringi’ package in R [33]. Alternating between each character and its corresponding number, an alphanumeric combination was concatenated. The letter–number combinations were then passed through a three-step script, with each step uniquely changing specific alphanumeric combinations. Substitutions of specific alphanumeric combinations to a new letter were designed to minimally change the phonetics of the resultant term. Due to the case sensitivity of R, the first letter of a term was never mutated. The ‘gsub’ function was run a final time to remove numbers and provide a list of mutated terms.

To avoid the generation of mutated terms with excessive repetitiveness, only the first occurrence of a repeating letter was kept and all long terms were truncated after the seventh character using the ‘stringr’ package in R [34]. Terms were subsequently shortened further to the last occurring vowel to prevent a hard consonant before the genus rank suffix ‘*-virus*’. We stipulated that each mutated name needed to be a minimum of five characters in length and contain at least two consonants and two vowels; these specific parameters required the initial starting list of terms to contain 1.5–2 times the number of genus names to be created while avoiding short terms. Finally, all newly created genus names were checked against the then-current ICTV taxonomy to ensure the uniqueness of the word stems of novel genera across all established taxon names (realms*→*species) and internet-searched to ensure produced terms were novel and not offensive (Fig. 5).

**Fig. 6.**
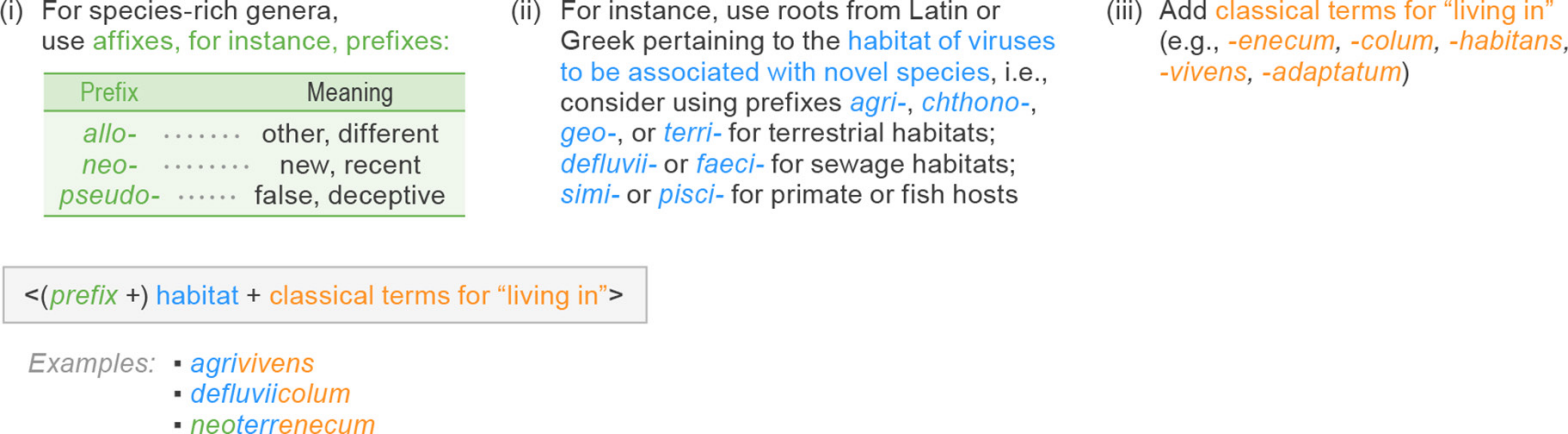
Example strategy for batch formation of virus species epithets.

The generation of novel species epithets was accomplished by devising a system to combine roots that describe different types of virus properties (Fig. 6). As initial species epithet elements, each epithet incorporated roots from Latin or Greek pertaining to the habitat of the discovered prokaryotic viruses. These roots were combined with second epithet elements reflecting classical terms for ‘living in’. For species-rich genera, we added a prefix to increase the number of epithets (see, for instance, https://www.bisdtx.org/cms/lib/TX02218757/Centricity/Domain/2450/Scientific%20Root%20Words.pdf for a broad list of affixes and Latin root words). Consequently, just a few dozen roots were used to generate more than 150 unique and linguistically well-formed species epithets. For detailed information on the etymology of all created species epithets, the interested reader is referred to the comments section of the Excel module of ratified ICTV TaxoProp 2020.095B.R.Leviviricetes; https://ictv.global/taxonomy/taxondetails?taxnode_id=202107200. Because the same set of species epithets may be used across distinct genera, this set of epithets enabled the creation of the 871 unique species names in combination with the newly created ones. Similar approaches can easily be programmed for any batch of genus/species names; by using various input parameters and diverse sets of affixes the number of created names can be increased as needed in an iterative manner. The combinatorial nature of this method thus permits the facile generation of Latinized binomial species names at a scale commensurate with even the most ambitious estimates for the rate of virus species establishment anticipated for the foreseeable future.

## Conclusion

Many of the initial reservations about the use of Latinized binomials for virus species names were based on the concern that converting thousands of names from English to a Latinized format would be complex and time-consuming. We hope this guide has demonstrated that converting existing names to and creation of new Latinized binomials is simpler and less arduous than imagined and is accessible to scientists without prior knowledge of Latin, i.e. that Latinization should not be equated with Latin translation. Latinization provides flexibility and assures a standardized and stable virus taxonomy that is comparable with that used by other branches of biology. Also, computational approaches make it possible to semi-automatically create hundreds of novel Latinized virus species names in bulk. A method similar to the one described here, applied to nearly 1000 prokaryotic virus species names, has been used to create close to 1 million new bacteria species names [28, 29], establishing the approach as a possible solution for species naming even if the number of species to be named increases massively [35]. While computational approaches may be iterative, as is the nature of software development, redundancy across platforms and within the methods provides stability and consistency for a solid yet flexible foundation upon which to build such models. Therefore, Latinized binomials offer a long-term viable alternative to other free-form naming approaches.

## References

[1] International Commission on Zoological Nomenclature (2012). International Code of Zoological Nomenclature.

[2] Linnaeus C (1753). Species plantarum.

[3] Parker CT, Tindall BJ, Garrity GM (2019). International Code of Nomenclature of Prokaryotes. Prokaryotic Code (2008 revision). Int J Syst Evol Microbiol.

[4] Turland NJ, Wiersema JH, Barrie FR (2017). International Code of Nomenclature for algae, fungi, and plants (Shenzhen Code) adopted by the Nineteenth International Botanical Congress Shenzhen, China, July 2017. Regnum Vegetabile 159.

[5] International Committee on Taxonomy of Viruses Executive Committee (2020). The new scope of virus taxonomy: partitioning the virosphere into 15 hierarchical ranks. Nat Microbiol.

[6] Calisher CH, Mahy BWJ (2003). Taxonomy: get it right or leave it alone. Am J Trop Med Hyg.

[7] Kuhn JH, Jahrling PB (2010). Clarification and guidance on the proper usage of virus and virus species names. Arch Virol.

[8] Van Regenmortel MHV (2003). Viruses are real, virus species are man-made, taxonomic constructions. Arch Virol.

[9] Walker PJ, Siddell SG, Lefkowitz EJ, Mushegian AR, Adriaenssens EM (2021). Changes to virus taxonomy and to the International Code of Virus Classification and Nomenclature ratified by the International Committee on Taxonomy of Viruses (2021). Arch Virol.

[10] Zerbini FM, Siddell SG, Mushegian AR, Walker PJ, Lefkowitz EJ (2022). Differentiating between viruses and virus species by writing their names correctly. Arch Virol.

[11] Gibbs A (2020). Binomial nomenclature for virus species: a long view. Arch Virol.

[12] Hull R, Rima B (2020). Virus taxonomy and classification: naming of virus species. Arch Virol.

[13] Postler TS, Clawson AN, Amarasinghe GK, Basler CF, Bavari S (2017). Possibility and challenges of conversion of current virus species names to *Linnaean binomials*. Syst Biol.

[14] Callanan J, Stockdale SR, Adriaenssens EM, Kuhn JH, Pallen M Rename one class (*Leviviricetes* - *formerly Allassoviricetes*), rename one order (*Norzivirales* - *formerly Levivirales*), create one new order (*Timlovirales*), and expand the class to a total of six families, 420 genera and 883 species. https://ictv.global/filebrowser/download/6027.

[15] Callanan J, Stockdale SR, Shkoporov A, Draper LA, Ross RP (2020). Expansion of known ssRNA phage genomes: From tens to over a thousand. Sci Adv.

[16] Edgar RC, Taylor J, Lin V, Altman T, Barbera P (2022). Petabase-scale sequence alignment catalyses viral discovery. Nature.

[17] Gregory AC, Zayed AA, Conceição-Neto N, Temperton B, Bolduc B (2019). Marine DNA viral macro- and microdiversity from pole to pole. Cell.

[18] Käfer S, Paraskevopoulou S, Zirkel F, Wieseke N, Donath A (2019). Re-assessing the diversity of negative strand RNA viruses in insects. PLoS Pathog.

[19] Krishnamurthy SR, Janowski AB, Zhao G, Barouch D, Wang D (2016). Hyperexpansion of RNA bacteriophage diversity. PLoS Biol.

[20] Li C-X, Shi M, Tian J-H, Lin X-D, Kang Y-J (2015). Unprecedented genomic diversity of RNA viruses in arthropods reveals the ancestry of negative-sense RNA viruses. Elife.

[21] Neri U, Wolf YI, Roux S, Camargo AP, Lee B (2022). Expansion of the global RNA virome reveals diverse clades of bacteriophages. Cell.

[22] Paraskevopoulou S, Käfer S, Zirkel F, Donath A, Petersen M (2021). Viromics of extant insect orders unveil the evolution of the flavi-like superfamily. Virus Evol.

[23] Shi M, Lin X-D, Chen X, Tian J-H, Chen L-J (2018). The evolutionary history of vertebrate RNA viruses. Nature.

[24] Shi M, Lin X-D, Tian J-H, Chen L-J, Chen X (2016). Redefining the invertebrate RNA virosphere. Nature.

[25] Starr EP, Nuccio EE, Pett-Ridge J, Banfield JF, Firestone MK (2019). Metatranscriptomic reconstruction reveals RNA viruses with the potential to shape carbon cycling in soil. Proc Natl Acad Sci U S A.

[26] Tisza MJ, Pastrana DV, Welch NL, Stewart B, Peretti A (2020). Discovery of several thousand highly diverse circular DNA viruses. Elife.

[27] Zayed AA, Wainaina JM, Dominguez-Huerta G, Pelletier E, Guo J (2022). Cryptic and abundant marine viruses at the evolutionary origins of Earth’s RNA virome. Science.

[28] Pallen MJ, Telatin A, Oren A (2021). The next million names for *Archaea* and *Bacteria*. Trends Microbiol.

[29] Pallen M, Alikhan N-F (2021). Naming the unnamed: over 45,000 *Candidatus* names for unnamed *Archaea* and *Bacteria* in the Genome Taxonomy Database. Biology.

[30] Callanan J, Stockdale SR, Adriaenssens EM, Kuhn JH, Rumnieks J (2021). *Leviviricetes*: expanding and restructuring the taxonomy of bacteria-infecting single-stranded RNA viruses. Microb Genom.

[31] International Committee on Taxonomy of Viruses (ICTV) (2022). ICTV Master Species List 2021.v3. Master Species Lists | ICTV.

[32] R Core Team (2020). R: A language and environment for statistical computing. https://www.R-project.org/.

[33] Gagolewski M (2020). stringi: THE String Processing Package for R. https://stringi.gagolewski.com/.

[34] Wickham H (2019). stringr: simple, consistent wrappers for common string operations. https://CRAN.R-project.org/package=stringr.

[35] Pallen MJ (2021). Bacterial nomenclature in the era of genomics. New Microbes New Infect.

